# Pulmonary functionality among workers of a Central Italy waste-to-energy plant: a retrospective study

**DOI:** 10.1186/s12995-019-0241-1

**Published:** 2019-07-04

**Authors:** L. Coppeta, A. Pietroiusti, S. Policardo, F. Mormone, O. Balbi, E. Tursi, S. Baldi, E. Plutoni, A. Torriero, A. Magrini

**Affiliations:** 1 Department of Occupational Medicine, University of Rome Tor Vergata, Occupational Health Service, Viale Oxford 81, 00133 Rome, Italy; 2 Department of Biomedicine and Prevention, University of Rome Tor Vergata, Rome, Italy

**Keywords:** Waste-to-energy, Industry-based, Retrospective cohort study, Waste incinerator, Pulmonary functionality, Spirometry, Respiratory function, Dust exposure

## Abstract

**Background:**

We are observing a growing trend towards the use of waste incineration in waste-to-energy (WTE) plants in Italy. Various authors started to investigate their potential health effects, but without univocal outcomes. The aim of this study is to assess whether or not main pulmonary function indexes could be decreased in a group of workers employed in a municipal solid WTE plant located in Central Italy, and if there’s a correlation between the levels of exposure to airborne pollutants and alterations in the pulmonary apparatus.

**Methods:**

The study was conducted with a retrospective cohort approach. We reviewed data from clinical records of 58 waste-to-energy plant workers undergoing annual health surveillance in the period 2010–2015. We considered the exposure to airborne dust and the main parameters of respiratory function (FVC, FEV1, Tiffeneau Index and FEF 25–75%) at time zero and after a period of 5 years. We divided our study population into two groups: low (< 1 mg/m3) and high (> 1 mg/m3) exposure. We estimated odds ratios (OR) with 95% confidence interval (95% CI) adjusted for potential confounders.

**Results:**

We observed a decrease in lung function parameters both in high and in low exposure group after a five-years exposure period. FEV1, FEV1/VC ratio and FEF 25–75% were worst in more exposed group, even if this difference resulted not significant at Wilcoxon test.

**Conclusions:**

Active employee in WTE plants is associated to a non-significant worsening in the main parameters of lung function after 5 years exposure. Clinical significant of these variations need to be assessed.

## Background

Urban solid waste disposal has become a widely regarded issue nowadays and this led to an implementation of various strategies in order to reduce the quantities of material deposited in landfill sites. A growing trend towards the use of waste incineration in waste-to-energy (WTE) plants has been observed in Italy in the last 10 years.

Simultaneously, this phenomenon originated concerns on potential health effects caused by this kind of waste disposal procedure. Various studies in literature revealed an increased risk for pulmonary, neoplastic and other diseases, in both incinerator plant workers and resident population nearby the plant area [1, 3].

Regarding occupational risk, there are concerns about environmental contamination from particulate matter, bio-aerosol, heavy metals, toxic chemicals (Be, Cd, Cr, Pb, Mn, Hg, Ni and V), and nanoparticles. In fact, the concentration of those substances is at particularly high levels in various areas of the plants. In published studies the levels of airborne dust ranged from 0.01 to 0.7 mg/m^3^ [4].

In particular, while on the one hand environmental assessments suggest evidence of possible carcinogenic, mutagenic, reproductive and pulmonary effects of occupational exposure, on the other hand evidence reported by limited epidemiological studies is contradictory [1–6].

The main lung effects investigated among workers are respiratory symptoms and pulmonary function [5–13]. In particular, the presence of functional respiratory alterations attributable to the current levels of exposure is controversial.

The main limitations in assessing the association between risk and occupational exposure in WTE plants are represented by an inaccurate and heterogeneous evaluation of the exposure levels [9].

Therefore, despite the size of the problem, studies concerning the evaluation of possible health effects caused by occupational exposure in WTE plants are scarce and mostly inadequate experimentally [14].

## Methods

Our study aimed to evaluate the pulmonary effects of exposure to airborne pollutants among a group of WTE workers employed in a central Italy plant, by investigating retrospectively the decrease in main pulmonary function indexes over a period of 5 years.

The initial study population consisted of 71 workers. We excluded data from 13 administrative employees without any exposure to airborne dust.

We finally included in the study records related to 58 male WTE workers who underwent annual medical screening in the period 2010–2015. The plant was located in the geographic area of Central Italy.

For privacy reasons, data were made anonymous through the insertion on two databases in Excel format, with an encrypted key available only to the investigators involved in the study. A database contained basic personal data (name and surname), while the other contained a personal code and all other variables, with the exception of first and last name.

People were considered eligible for the study if they had worked in the plant for almost 5 years. The exclusion criteria were the presence of systemic or immunological disorders, the presence of neoplasia, a previous diagnosis of asthma, emphysema, or interstitial lung disease [14].

At least three measurements of personal exposure to total dust were available during the examined period; the measurement was calculated as the mean level of exposure (mg/m3 during 8 h of work).

Two levels of exposure were established a priori: low (< 1 mg/m3) and high (> 1 mg/m3). Those limits were chosen on the basis of current guidelines regarding the risk of respiratory disorders after occupational exposure to total dust [15, 16]: according to these guidelines, the threshold limit for generic dust is 10 mg/m3, and one-tenth of the threshold limit is considered as the “attention level” beyond which adverse respiratory effects cannot be excluded.

Thoracic fraction (median size 11.6 μm diameter) was considered in this study because it is the mass fraction that can reach the medium airways [17]. The level of the dust exposure was assessed by the gravimetric method, which is based on the collection of the thoracic fraction samples on filters made of poly-vinyl chloride at a flow rate of 2.2 l/min.

Lung function test had been performed every year: we collected data from the first and the last year of the observation period (year 2010 and 2015 respectively).

All the workers enrolled in the study had performed a full ventilatory manoeuvre, both slow and forced acquisition of volume-time and flow-volume loop.

The measurement of lung volumes and flows had been assessed using a flow-based spirometer (SensorMedics, Vmax 20) according to the guidelines of the ATS (American Thoracic Society)-ERS (European Respiratory Society) 2005 workforce. At last, three consecutive acceptable trials had been performed for each subject [18].

From spirometry we collected the following parameters: Forced Vital Capacity (FVC); forced expiratory volume in the first second (FEV 1 or FEV); Tiffeneau Index (FEV1/VC); Peak Expiratory Flow (PEF); forced expiratory flow at 25% of FVC (FEF25%); forced expiratory flow at 50% of FVC (FEF50%); forced expiratory flow at 75% of FVC (FEF75%); forced expiratory flow between 25 and 75% of FVC (FEF25–75%).

Decrease of lung function parameters after the 5 years period was recorded. Worsening in FEV1, FEV1/VC ratio (Tiffeneau Index) and in FEF 25–75% were considered as a possible dust exposure effect [19]. Decrease in lung function test was calculated both as quantitative and as dichotomous (yes or not) variable. All of the workers were investigated for main potentially confounding variables, such as age (more or less than 40 years old) and smoking habit. Gender influence was not evaluated since no female worker was employed in the plant.

No sample size study was conducted before and all eligible subjects were included in the study. The data were analyzed with STATA 11 statistical package. We used Chi Squared test for dichotomous variables and Mann-Whitney U test and Wilcoxon test for the continuous variables (FEV1, FVC, FEF25–75%, FEV1/VC) since they showed non-normal distribution. Having a decline in FEV1/VC was the dependent variable of a binary logistic regression model, with age, smoking habits and dust exposure as independent variables [19].

The study was approved by the ethics committee of our institution.

## Results

We analyzed data from 58 male WTE workers. Mean age was 46.7 ± 8.0 (range 35–59) years. Mean exposure level (thoracic dust fraction) was 0.76 ± 0.9 (range 0.01–2.35) mg/m3. According to our exposure criteria, 37 workers were classified as having low exposure and 21 as having high exposure. Mean seniority was 7.9 ± 2.2 years of employment. The main characteristics of the study population are shown in Table 1.

**Table 1:** Main characteristics of the study population

	*Low exposure*_*a*_	*High exposure*_*b*_	*p value (χ* ^*2*^ *test)*
*Number (%)*	37 (63.8)	21 (36.2)	
*Exposure thoracic dust fraction (mg/m3)*	0.15	1.81	
*Age (mean; yrs)*	47.5	45.4	
*Age class*			
*< 40 years*	16 (57.1)	12 (42.9)	0.31
*> 40 years*	21 (70.0)	3 (30.0)	
*Work seniority*			
*< 10 years*	27 (57.4)	20 (42.6)	0.04
*> 10 years*	10 (90.9)	1 (9.1)	
*Smokers (%)*			
*Yes*	15 (57.7)	11 (42.3)	0.38
*Not*	22 (68.7)	10 (31.3)	

_*a*_ *Defined as < 1 mg/m3*

_*b*_ *Defined as > 1 mg/m3*

Regarding the role of possible confounders, smokers were found with increased frequency among the low-exposure workers in comparison to those with high exposure (57.7% versus 42.3%, p = ns); also exposure was lower among workers with work seniority> 10 years (57.4% versus 42.6%, with a significant *p* = 0.04) and below the age of 40 (57.1% versus 42.9%, p = ns).

While tested with Wilcoxon test, significant lowering in all of lung function parameters was observed both in high and in low exposure group after a five-years exposure period (*p* < 0.01). Fall in FEV1, FEV1/VC ratio and FEF 25–75% were higher in more exposed group even if this difference resulted not significant at Mann-Whitney U-test (Table 2).

**Table 2:** Lung function parameters in low exposure and high exposure

	*Mean*	*Difference*	*S.D.*	*S.E.*	*95% C.I.*	p value*
*FEV*_*1*_ *low exposure*	0.22	− 0.07	0.43	0.06	(0.13–0.35)	0.34
*FEV*_*1*_ *high exposure (Liters)*	0.29					
*FVC low exposure*	0.40	− 0.12	0.50	0.07	(0.32–0.58)	0.65
*FVC high exposure (Liters)*	0.52					
*FEV*_*1*_ */VC low exposure*	0.16	− 0.12	0.41	0.05	(0.10–0.31)	0.93
*FEV*_*1*_ */VC high exposure (Percentage)*	0.28					
*FEF*_*25–75*_ *% low exposure*	0.19	− 0.05	0.41	0.05	(0.10–0.31)	0.43
*FEF*_*25–75*_ *% high exposure (Liters)*	0.24					

[*S.D.* Standard Deviation, *S.E.* Standard Error, *95% C.I.* Confidence Interval]

* Mann Whitney U Test

Reduced value of FEV1/FVC was found in 12/58 (20.7%) of study population after a 5 years period. A non-significant association between this parameter and level of exposure was found even considering the potential confounding role of smoking habit, age and seniority (Table 3).

**Table 3:** Logistic regression analysis

	*Decline in FEV1/VC*			
	*O.R.*	*95% C.I.*	*p value*	
*Smoking habit*	1.82	(0.49–6.85)	0.37	0.89
*Seniority > 10 years*	2.39	(0.44–12.88)	0.31	1.02
*Age > 40 years*	1.57	(0.41–6.01)	0.51	0.66
*High exposure*	2.67	(0.62–11.48)	0.19	1.32

[*O.R.* Odds Ratio, *95% C.I.* Confidence Interval]

## Discussion

Lung function tests evaluation in the work setting should be part of a workplace respiratory health surveillance program among workers exposed to airborn dust [20]. Despite the community concerns about potential health effects of WTE plants, only few studies evaluated the lung function changes related to dust exposure among workers employed in those setting. Our study shows a decrease in airflow after a 5 years working period among WTE employees. The lowering in respiratory parameters was observed both in high and in low levels of exposure.

Since a physiological fall in lung function index was expected in the same period due to population ageing, the main result correlating with the possible effects of dust exposure is differential decrease between groups having different levels of exposure, despite this difference came out to be not significant at Wilcoxon test.

We observed a uniform trend to a worsening of all respiratory parameters in relation to the levels of exposure, including FEF25–75%, that has been reported to be more sensitive than FEV1, and FEV1/VC ratio, in order to predict the development of clinically evident airflow obstruction associated with air pollution [21, 22] although their potential weakness is represented by its relatively high day-to-day variability [19].

Among our population smoking habit was related to higher risk of fall in FEV1/VC (although not statistically significant) suggesting a synergetic effect of exposure to dust and smoking.

Despite large epidemiological studies are lacking, results are partially consistent with the few published studies regarding the onset of airflow reduction in workers exposed to incinerator dust [19]. Bresnitz et al. [6] studied a cohort of 86 actively employed male workers of Philadelphia WTE plant; workers were divided into high and low exposure groups, and no statistically significant differences in main pulmonary function index were found between the two groups, whereas changes in pulmonary function were reported to be related only to smoking status. In a transversal study carried out for 102 male incinerator workers, a significant relationship between exposure and the decrease of several pulmonary parameters was observed [5].

Taking in account differences in chemical composition of airborne dusts some data emerging from occupational cement dust exposure survey can be compared with results of our study. In a large prospective study conducted among cement workers increased odds ratios for symptoms and airflow limitation and 270 mL fall in FEV1 was observed in the highest quartile of exposure. Those results are consistent with the 285 mL decrease in FEV1 that we found in the high exposure group, but we failed to reach statistical significance. In an Italian study among 36 cement workers followed for 11 years, a decline of 340 mL in FEV1 was observed. This fall was equal to an annual decline of 31 mL [23]. In a study from former Yugoslavia, a significant decline in FEV1/FVC was reported in an 8-year follow-up of a large group of cement workers.

In a recent review lung function tests have shown to be able to identify novel occupational respiratory diseases such as flavoring-related lung disease [24].

Possible limitations of our study are represented by the small size of our sample, the absence of a control group and by the selection mechanisms that may possibly have resulted in a healthy worker effect, commonly reported in previous studies regarding FEV1 and industrial exposure [5, 25].

The natural longitudinal lowering of lung function parameters may accelerate with increasing age [26, 27]. To evaluate a possible age effect, we tested between age > 40 years (yes/no), smoking habit and exposure in a multivariate analysis. The association remained negative after considering age and smoke confounding effect.

Another limitation of our study was that both low and high exposure workers were men, so the findings cannot be extrapolated for women. Waste incinerator dust exposure should be frequently found among women and it is possible that dust exposure in other occupational and extra-occupational settings may have different effects on lung function among women and younger subjects.

## Conclusions

In our study, exposure to WTE plant airborne dust is associated to a non-significant worsening in the main parameters of lung function after 5 years exposure. These impairments are mild, according to ATS/ERS 2005 criteria [19], and the levels of airborne pollutants identified were constantly under the occupational threshold limit value. Clinical significant of these variations need to be assessed.

Further studies are necessary to confirm the findings of our study.

## Data Availability

The datasets used and/or analyzed during the current study are available from the corresponding author on reasonable request.

## Abbreviations

ATS: American Thoracic Society
CIC: Confidence Interval
ERS: European Respiratory Society
FEF: Forced expiratory flow
FEV1: Forced expiratory volume in the first second
FVC: Forced Vital Capacity
OR: Odds ratios
PEF: Peak Expiratory Flow
VC: Vital Capacity
WTE: Waste-to-energy
